# Analysis of conditional gene deletion using probe based Real-Time PCR

**DOI:** 10.1186/1472-6750-10-75

**Published:** 2010-10-15

**Authors:** Britta Weis, Joachim Schmidt, Frank Lyko, Heinz G Linhart

**Affiliations:** 1 Division of Epigenetics, German Cancer Research Center, Heidelberg, Germany; 2 Division of Translational Oncology, German Cancer Research Center, Heidelberg, Germany; 3 Department of Gastroenterology and Hepatology, University Hospital Freiburg, Hugstetter Str. 49, 79106 Freiburg, Germany

## Abstract

**Background:**

Conditional gene deletion using Cre-lox recombination is frequently used in mouse genetics; however recombination is frequently incomplete, resulting in a mixture of cells containing the functional (2lox) allele and the truncated (1lox) allele. Conventional analysis of 1lox/2lox allele ratios using Southern Blotting is time consuming, requires relatively large amounts of DNA and has a low sensitivity. We therefore evaluated the utility of Real-Time PCR to measure 1lox/2lox allele ratios.

**Results:**

We show that SYBR Green based Real-Time PCR analysis of 1lox/2lox allele ratios can generate erroneous peaks in the melting curve that are possibly caused by alternate hybridization products promoted by the palindromic loxP sequence motif. Since abnormal melting curves frequently contribute to dismissal of SYBR Green based data, we developed a convenient method with improved specificity that avoids such erroneous signals. Our data show that probe based Real-Time PCR, using a universal probe directed against the loxP site, can accurately detect small differences in 1lox/2lox allele ratios. We also validated this method in Fabpl^4× at -132^-Cre transgenic mice, measuring 1lox/2lox allele ratios that are in agreement with published data. Our Real-Time PCR protocol requires the use of one probe only for all reactions. Also the universal probe established in our assay is generally applicable to any experiment analyzing Cre-lox recombination efficiency, such that only primer sequences have to be adapted.

**Conclusions:**

Our data show that 1lox/2lox allele ratios are detected with high accuracy and high sensitivity with Real-Time PCR analysis using a probe directed against the loxP site. Due to the generally applicable probe the assay is conveniently adapted to all models of Cre-lox mediated gene deletion.

## Background

Gene knock out technology is widely used to study gene function *in vivo*, however in some cases constitutive gene deletion is either lethal or deleterious for embryonic development precluding analysis of the adult organism. In these instances it is necessary to induce gene deletion in a tissue specific or time specific manner. Tissue specific gene knock out models are most often constructed using the Cre-lox recombination system. For this, the sequence of interest is flanked by loxP sites and conditional deletion is achieved by tissue specific expression of the Cre recombinase enzyme [1-3]. Due to variability in Cre recombinase expression however, deletion of the gene of interest is frequently incomplete. In such cases the tissue of interest would consist of a mixture of cells containing either the functional allele (2lox) or the truncated allele (1lox) [4,5]. Clearly, the ratio of 2lox containing cells to 1lox containing cells can have a profound impact on the outcome of the experiment. Also, in many cases the ratio of these two cell types can change over time, particularly in regenerating tissues, causing additional variability in the phentotype. In the worst case the presence of cells with the functional 2lox allele can fully mask the effect of the gene deletion. For example if tumors are detected following the conditional deletion of a tumor relevant enzyme it is possible that such tumors are formed by residual 2lox cells [6]. Therefore to better understand the phenotype of such experiments it is imperative to accurately measure the relative presence of 1lox and 2lox alleles at various time points of the study.

The conventional approach to determine the ratio of 1lox to 2lox alleles is Southern Blotting [7]. This method however is time consuming, requires large amounts of DNA (frequently 5 μg or more) and has a relatively low sensitivity and low dynamic range. Therefore alternative methods such as competitive endpoint PCR [6] and eMLPA [8] have also been used. However since many laboratories routinely use Real-Time PCR for quantitative RNA or DNA sequence analysis we decided to develop a Real-Time PCR based assay to measure 1lox/2lox allele ratios.

We here present a universally applicable probe based Real-Time PCR protocol that allows accurate quantification of 1lox/2lox allele ratios in genomic DNA. This assay is conveniently adaptable to all models using Cre-lox mediated gene recombination. In addition we demonstrate and discuss potential pitfalls in particular when using SYBR Green based protocols.

## Results

To evaluate the role of DNA methyltransferase 3a (Dnmt3a) in tumorigenesis we induced conditional deletion of Dnmt3a (Dnmt3a^(2lox/2lox)^) in intestinal epithelial cells of APC^(Min/+) ^mice using a Cre recombinase transgene under control of the modified fatty acid binding protein promoter (Fabpl^4× at -132^-Cre^(+/-)^) [6,9]. As published previously, Fabpl^4× at -132^-Cre mediated gene deletion in epithelial cells of the intestinal tract is incomplete [5]. It was therefore necessary to quantify the ratio of the functional Dnmt3a 2lox allele (2lox) and the truncated Dnmt3a 1lox allele (1lox) in the intestinal mucosa and in intestinal tumors of transgenic mice. To allow processing of large sample numbers with small amounts of DNA we tested whether 1lox/2lox allele ratios could be measured using a Real-Time PCR based assay

### Testing of SYBR Green based Real-Time PCR for analysis of conditional gene deletion

**Figure 1:**
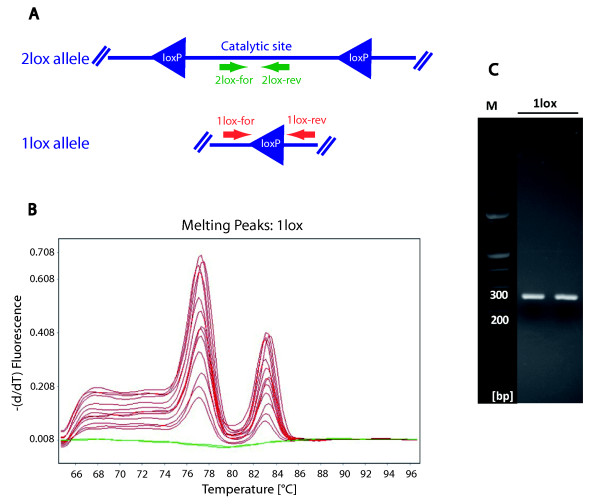
**SYBR Green based Real-Time PCR of loxP containing sequences yields an aberrant melting curve**. A Strategy for primer placement: Primers specific for the 2lox allele are placed within the loxP flanked genomic region and primers specific for the 1lox allele are placed into unique genomic sequences surrounding the remaining loxP site of the truncated allele. **B **Representative melting curve analysis of the 1lox reaction (the figure shows different primer concentrations measured in triplicates). This reaction consistently produced a double peak possibly due to the palindromic loxP sequence that can promote annealing of sense/sense and antisense/antisense homodimers. The same was observed when using alternative primer pairs, different annealing temperatures, different DNA concentrations and even the gel purified 1lox PCR product. **C **Gel electrophoresis of the 1lox Real-Time PCR reaction showed a single band, confirming that the double peak in the melting curve is not caused by the presence of an additional nonspecific amplicon.

**Figure 2:**
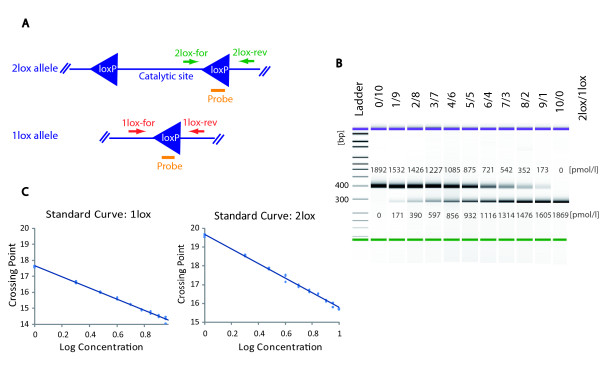
**Probe based Real-Time PCR analysis of conditional gene deletion**. A Strategy for probe and primer placement: The probe (UP #69, Roche Applied Science) is complementary to the loxP site and was used for both reactions. For analysis of the 2lox allele, primers were placed in unique genomic sequence surrounding the 3' loxP site. For the 1lox reaction primers were placed in unique genomic sequence surrounding the loxP site of the truncated allele. **B **Validation of Real-Time PCR standards using capillary electrophoresis: DNA fragments containing the 1lox and 2lox target sequence respectively were mixed to generate standards with molar ratios ranging from 10/0 to 0/10. Capillary electrophoresis (measured molarities inscribed in the gel image) confirmed that the standards contained the expected ratios of 1lox and 2lox specific sequences. **C **Real-Time PCR efficiency is not affected by the presence of the alternative allele: 1lox (left graph) and 2lox (right graph) specific Real-Time PCR reactions were conducted using the mixed standards shown in section B. Both reactions show a linear correlation between Crossing point (Cp) and log concentration values indicating that the reaction efficiency is not affected by the presence of the other allele.

To allow analysis of gene deletion using SYBR Green based Real-Time PCR we designed primers allowing specific amplification of the 1lox and 2lox allele repectively. As illustrated in **Figure **1A positioning of the 2lox specific primers is comparatively easy because they can be placed anywhere within the potentially deleted region. However it has to be considered that in some cases the excised gene fragment can persist, particularly in non-proliferating tissue after initiation of Cre expression [8]. Therefore in such cases - in tissues with low mitotic activity and shortly after induction of Cre expression - 2lox primer placement in analogy to **Figure **2A might be preferable. When designing 1lox specific primers, care has to be taken to place them within unique genomic sequences flanking the remaining loxP site of the truncated 1lox allele (**Figure **1A). However, when following this approach to quantify conditional Dnmt3a deletion in our mice we noted the continuous presence of a double peak in the melting curve of the 1lox reaction (**Figure **1B). This double peak persisted even when choosing different annealing temperatures, multiple alternative primer pairs for the 1lox reaction, different primer concentrations and different DNA concentrations. Since the presence of two peaks in the melting curve of SYBR Green based Real-Time PCR assays frequently indicates the presence of two different amplicons we analyzed the resulting PCR product using both gel electrophoresis (**Figure **1C) and capillary electrophoresis (data not shown). Surprisingly, although we had observed two peaks in the melting curve analysis, we could identify only one distinctive PCR product. Furthermore when we size selected the PCR product using gel purification to exclude primers and alternative products and repeated the melting curve analysis we reproduced the same double peak (data not shown), suggesting that the amplified sequence itself was responsible for this aberrant signal. A possible explanation for this observation is that the palindromic loxP sequence motif could serve as a core motif that promotes annealing of sense/sense and antisense/antisense strands or possibly the formation of hairpins. Such hybridization products/hairpins would have a lower melting temperature than antisense/sense hybrids and therefore contribute to a second peak in melting curve analysis. Since the melting curve is critical for quality control in SYBR Green assays, data with an aberrant double peak are frequently excluded from further analysis. Our example illustrates that SYBR Green based assays are not only limited by possible amplification and measurement of nonspecific PCR products but are also potentially affected by alternate re-annealing or secondary structures of the specific PCR product itself. In agreement with our own data, difficulties with SYBR Green based analysis of 1lox/2lox allele ratios have also been reported independently [8].

To resolve such potential difficulties of SYBR Green based analysis of loxP containing sequences we therefore developed an alternative assay to quantify 1lox/2lox allele ratios using a probe based Real-Time PCR approach. Our aim was to establish a universal probe that could be used for all reactions and models of Cre-lox mediated gene deletion such that only the flanking primer sequences need to be adapted. Such an assay would combine the convenience of a SYBR Green based assay with the specificity of a probe based assay.

### Testing of probe based Real-Time PCR for analysis of conditional gene deletion

**Figure 3:**
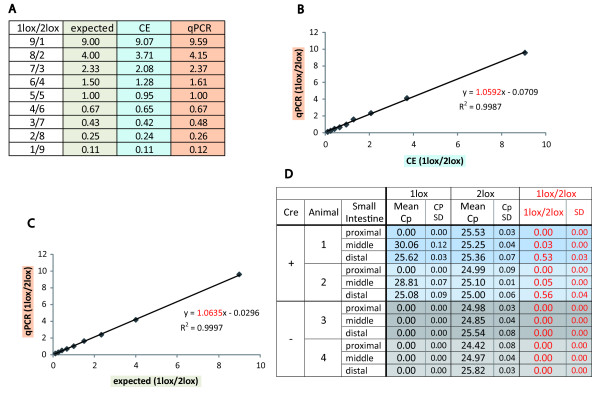
**Probe based Real-Time PCR measures 1lox/2lox allele ratios with high accuracy**. A, B, C Real-Time PCR analysis was conducted using standards with variable 1lox/2lox allele ratios. The resulting data were compared with expected ratios (**C**) and with ratios measured by capillary electrophoresis (CE), (**B**). As summarized in **A **the Real-Time PCR data closely reflected the expected data and the CE data. **D**: Deletion of Dnmt3a was measured in intestinal epithelial cells of mice homozygous for the Dnmt3a 2lox allele with Fabpl^4× at -132^-Cre transgene (Cre^+^) and without Fabpl^4× at -132^-Cre transgene (Cre^-^). In agreement with previous studies using the same Fabpl^4× at -132^-Cre strain, Cre^+ ^mice showed low loop out efficiency in the proximal small intestine (1lox/2lox ratio of 0) and higher loop out efficiency in the distal small intestine (1lox/2lox approximately 0.5). Data from animal 1 middle and animal 2 middle are slightly out of standard range. As expected the 1lox allele was not detectable in Cre^- ^mice (mean = Cp of three replicates, SD = standard deviation).

To establish a probe based assay for the 1lox and 2lox reaction respectively we selected a hydrolysis probe (UP #69, Roche Applied Science) that specifically binds to the loxP site itself and designed primers flanking the loxP site as illustrated in **Figure **2A. The use of a probe covering the loxP site (UP #69) allowed us to use the same probe for both the 1lox and 2 lox reaction. To generate allele specific standards that would allow us to validate the assay and test its accuracy we cloned 1lox and 2lox specific PCR products into a TA vector and verified resulting clones by sequencing. To generate standards for Real-Time PCR we then released allele specific fragments by restriction digest and mixed the fragments at variable molar ratios. The resulting 1lox/2lox standards were verified using capillary electrophoresis, which confirmed the expected ratios (**Figure **2B and Figure 3A).

To determine the relative efficiency of the 1lox and 2lox specific Real-Time PCR reaction and to detect possible interference between the two alleles we conducted Real-Time PCR using 1:100 dilutions of the above mentioned fragment mixtures. As illustrated in **Figure **2C we observed a linear correlation between the Crossing point (Cp) and Log concentration data for both reactions, with all data points close to the curve (r^2 ^> 0.99), indicating that the efficiency of both reactions was not affected by variable admixtures of the other allele. To further test the accuracy of the Real-Time PCR assay we conducted quantitive PCR, using incremental standards ranging from 9/1 to 1/9 and calculated allele ratios from the measured data. As summarized in **Figure **3A the 1lox/2lox allele ratios as determined by the probe based Real-Time PCR assay closely reflected allele ratios measured by capillary electrophoresis (**Figure **3B) and the expected allele ratios (**Figure **3C).

### Testing of probe based Real-Time PCR for analysis of Cre-lox recombination in Fabpl^4× at -132^-Cre transgenic mice

After establishing the accuracy of the method we measured conditional deletion of Dnmt3a in intestinal epithelial cells of Fabpl^4× at -132^-Cre transgenic mice. For this we harvested intestinal epithelial cells from the proximal, middle and distal third of the small intestine of Dnmt3a^(2lox/2lox)^, Fabpl^4× at -132^-Cre^(+/-) ^mice and measured 1lox/2lox allele ratios using Real-Time PCR. As shown in **Figure **3D we detected a low deletion efficiency in the proximal small intestine and increasing deletion efficiency in the distal small intestine, with the 1lox/2lox allele ratio varying from 0 to approximately 0.5 respectively. Importantly the data of the two Cre^+ ^mice were highly similar and the variation within the sample replicates was very low, confirming the reproducibility of the method **(Figure **3D). Also, these results are in good agreement with published data on the same Fabpl^4× at -132^-Cre strain using Northern Blotting and Immunohistochemistry [10], which further confirms the utility of the assay.

## Discussion

Real-Time PCR is clearly preferable to Southern Blotting when attempting to quantify conditional gene deletion: Real-Time PCR offers a higher sensitivity, requires significantly smaller DNA samples and requires much less processing time. This is particularly useful when allele ratios have to be measured in small samples at several time points, such as in conditional deletion experiments involving hematopoetic stem cells [11]. Surprisingly, only one study formally evaluated this technical approach, however without testing the ability of the method to detect small changes in allele ratios [12].

In principle either SYBR Green based protocols or probe based protocols can be used for Real-Time PCR. SYBR Green based assays are very popular because of the relatively low cost and the convenient implementation requiring primer design only.

However, as illustrated by our example, it appears that SYBR Green based protocols for quantification of conditional gene deletion, are potentially complicated by the palindromic sequence of the loxP site. We show that PCR products containing this sequence, such as the 1lox specific PCR product, can generate double peaks during melting curve analysis, likely due to alternate product hybridization, which can complicate quality control of the reaction. In such cases it is uncertain whether the double peak reflects the presence of an additional nonspecific PCR product or not. We therefore suggest that it is generally preferable to use a probe based Real-Time PCR approach, which avoids such erroneous signals and offers improved specificity. The hydrolysis probe established in our protocol (UP #69, Roche Applied Science), that is used for analysis of both alleles, recognizes the loxP site itself and is therefore universally applicable to any assay measuring 1lox/2lox allele ratios. To adapt the assay to other conditional alleles it is therefore only necessary to place primer pairs in the unique genomic sequence flanking the loxP sites. Our data support the notion that such probe based Real-Time PCR analysis allows reproducible and accurate measurement of 1lox/2lox allele ratios with small amounts of DNA and is therefore a highly attractive alternative to conventional Southern Blotting.

## Conclusions

Our results suggest that probe based Real-Time PCR can accurately detect small differences in 1lox/2lox allele ratios with improved specificity. Our protocol requires the use of one probe only for all reactions. This probe is directed against the loxP site itself and is therefore universally applicable to any assay analyzing Cre-lox recombination efficiency, such that only the primer sequences have to be adapted. Our assay therefore combines the convenience of SYBR Green based analysis with the specificity of probe based analysis and is applicable to all models of Cre-lox mediated conditional gene deletion.

## Methods

### Preparation of the 1lox/2lox standard

1lox and 2lox specific PCR products (1lox-forward primer: GCCGGCTTTTCCTCA GACAGTGGAGATAGC, 1lox-reverse primer: CCTGTGTGCAGCAGACACTTCT TTGGCGTC; 2lox-forward primer: CCTCTGGGGATTAAACTCTTGGCCAGCCC, 2lox-reverse primer = 1lox-reverse primer) were generated using genomic DNA from Dnmt3a^(2lox/2lox)^, Fabpl^4× at -132^-Cre^(+/-)^, APC^(Min/+) ^transgenic mice. PCR products were then purified using agarose gel-electrophoresis followed by gel extraction (QIAGEN Minielute Gelextraction Kit) and cloned into TA vectors (TOPO-cloning Kit, Invitrogen). Minipreps were prepared from individual colonies resulting from both 1lox and 2lox ligations and verified by sequencing. Plasmids with verified sequences were digested with EcoRI to release fragments containing the 1lox and 2lox specific inserts, respectively. Following gel electrophoresis and gel extraction the concentration of resulting 1lox and 2lox fragments were measured using capillary electrophoresis (Agilent, High sensitivity DNA Assay) and 1lox-2lox mixtures were prepared at variable molar ratios (1/9, 2/8, 3/7, 4/6, 5/5, 6/4, 7/3, 2/8, 1/9). The 1lox/2lox ratios of these final mixtures were verified again using capillary electrophoresis.

### Probe based Real-Time PCR

The hydrolysis probe based Real-Time PCR reaction was performed as follows: 1lox- and 2lox reactions were conducted separately in 10 μl reactions containing 0.25 μM each of forward and reverse primers (1lox-forward primer: TAATCCCAGCACTGC ACTCA, 1lox-reverse primer: TTCTTTGGCGTCAATCATCA; 2lox-forward primer: CCTCTGGGGATTAAACTCTTGGCCAGCCC, 2lox-reverse primer: CCTGTGTGC AGCAGACACTTCTTTGGCGTC), 0.05 μl Universal Probe #69 (Roche Applied Science), LightCycler 480 Probes Master Mix (Roche Applied Science) and 50ng genomic DNA or 4.3pg of the 1lox/2lox standard. The cycling conditions were as follows: Preincubation at 95°C for 10min, then 45 cycles of 95°C for 10sec, 58°C for 45sec and 72°C for 1sec. Each reaction was measured in triplicates. For data analysis we used "LightCycler(r) 480 Software release 1.5.0 SP3" (Roche Applied Science) and the "Advanced Relative Quantification" module where the crossing points (Cp = Ct) were determined using the "2nd Derivative Maximum Method". The mentioned module quantifies 1lox signal (**T**, Target) relative to 2lox signal (**R**, Reference), considering the respective amplification efficiencies (**E**) using the following formula: **E_T_^CpT ^/E_R_^CpR^**. The efficiencies were determined using the slope of the respective standard curve (E = 10^-1/slope^) with Cp values plotted against the logarithm of different dilutions. As dilution standards we used the incremental 1lox/2lox standard mixtures (10 standards, 10% dilution steps), in other words the samples themselves were used for calculation of reaction efficiencies. As a final control the PCR product was analyzed using agarose gel electrophoresis.

### SYBR Green based Real-Time PCR

Real-Time PCR was performed using equipment of Roche Applied Science (LightCycler 480 SYBR Green I Master, Light Cycler 480). 1lox and 2lox-reactions were conducted separately in 10 μl volume containing 50ng DNA and 0.25 μM each of forward and reverse primers (1lox-forward primer: TAATCCCAGCACTGCACTCA, 1lox-reverse primer: TTCTTTGGCGTCAATCATCA; 2lox-forward primer: CCGAT GCAGACAGCCTCAGC, 2lox-reverse primer: CTTGTCACTAACGCCCATGG CCA). The following cycling conditions were chosen: Preincubation at 95°C for 10min, then 45 cycles of 95°C for 10sec, 60°C for 15sec and 72°C for 30sec. Each reaction was measured in triplicates. Data analysis was executed in analogy to Probe based Real-Time PCR. As a final control the PCR product was analyzed using agarose gel electrophoresis.

### Animals

The Dnmt3a^(2lox/2lox)^, Fabpl^4× at -132^-Cre^(+/-)^, APC^(Min/+) ^transgenic mice were generated as previously described for Dnmt3b^(2lox/2lox)^, Fabpl^4× at -132^-Cre^(+/-)^, APC^(Min/+) ^mice [5,6]. Briefly the conditional Dnmt3a allele contains loxP sites located in intron 17 and intron 20 of the endogenous Dnmt3a allele. Cre mediated excision results in deletion of exons 18-20, resulting in deletion of the catalytic domain. For a detailed description of the Dnmt3a ^(2lox/2lox) ^mice see [9], for details on Fabpl^4× at -132^-Cre^(+/-) ^mice see [5].

No animal experiments were conducted with the intention of generating material for the present study. All mouse tissues and DNA analyzed in the present study were harvested for the purpose of an alternative, ongoing project analyzing the role of DNA Methyltransferase 3a in mouse tumorigenesis. All animal experiments of that project were approved by the MIT Department of Comparative Medicine, Boston, USA and were executed according to the institutional guidelines.

### Harvesting of epithelial cells of the small intestine

Intestinal epithelial cells were harvested as described previously [13]. Briefly, the small intestine was dissected from mice, divided into three thirds of equal length (proximal, middle, distal), rinsed and incubated in 20 ml harvesting buffer (3 mmol/L ethylenediaminetetraacetic acid (EDTA) plus 0.5 mmol/L dithiothreitol in PBS) at 37°C, rotating at 250 rpm for 30 min. Following incubation the intestinal tube was removed and floating epithelial cells centrifuged at 200×g and 4°C for 4 min. After removal of the supernatant, cell pellets were frozen at -80°C.

### DNA-Isolation

DNA of the harvested small intestine epithelial cells was isolated using the QIAGEN DNeasy Kit using the manufacturer's protocol.

## Authors' contributions

B.W. conducted experiments, data analysis and co-wrote the manuscript, J.S. contributed to experiments and data analysis, F.L. contributed to experimental planning, data interpretation and corrected the manuscript, H.L. conceived the project, contributed to experimental planning, data analysis and interpretation and co-wrote the manuscript. All authors read and approved the final manuscript.
