# Analysis of conditional gene deletion using probe based Real-Time PCR

**DOI:** 10.1186/1472-6750-10-90

**Published:** 2010-12-21

**Authors:** Britta Weis, Joachim Schmidt, Frank Lyko, Heinz G Linhart

**Affiliations:** 1 Division of Epigenetics, German Cancer Research Center, Heidelberg, Germany; 2 Division of Translational Oncology, German Cancer Research Center, Heidelberg, Germany; 3 Department of Gastroenterology and Hepatology, University Hospital Freiburg, Hugstetter Str. 49, 79106 Freiburg, Germany

## Abstract

Following publication of this article [1] the authors noticed that an incorrect probe reference was cited on page 3, 4, 5 and 6 ("UP #69, Roche Applied Science"). The correct probe that was used for the 1lox/2lox allele ratio analysis in the paper is as follows

Probe for 1lox/2lox allele quantification:

5'-**6-FAM-atAaCtTCgtatagCATaCattatac-BHQ-1 **-3'

(**uppercase letters = LNA bases**)

Manufacturer: EUROGENTEC, Seraing, Belgium

All other information and reaction conditions in the paper are correct as stated.
